# Spatial distribution patterns of soil mite communities and their relationships with edaphic factors in a 30-year tillage cornfield in northeast China

**DOI:** 10.1371/journal.pone.0199093

**Published:** 2018-06-28

**Authors:** Jie Liu, Meixiang Gao, Jinwen Liu, Yuxi Guo, Dong Liu, Xinyu Zhu, Donghui Wu

**Affiliations:** 1 College of Earth Science, Jilin University, Changchun, China; 2 Key Laboratory of Wetland Ecology and Environment, Northeast Institute of Geography and Agroecology, Chinese Academy of Sciences, Changchun, China; 3 College of Geographical Sciences, Harbin Normal University, Harbin, China; 4 Jilin Academy of Agricultural Sciences, Changchun, China; 5 College of Environment and Planning, Shangqiu Normal University, Shangqiu, China; 6 School of Environment, Northeast Normal University, Changchun, China; 7 Key Laboratory of Vegetation Ecology, Ministry of Education, Northeast Normal University, Changchun, China; 8 Jilin Provincial Key Laboratory of Animal Resource Conservation and Utilization, Northeast Normal University, Changchun, China; Pacific Northwest National Laboratory, UNITED STATES

## Abstract

Spatial distribution is an important topic in community ecology and a key to understanding the structure and dynamics of populations and communities. However, the available information related to the spatial patterns of soil mite communities in long-term tillage agroecosystems remains insufficient. In this study, we examined the spatial patterns of soil mite communities to explain the spatial relationships between soil mite communities and soil parameters. Soil fauna were sampled three times (August, September and October 2015) at 121 locations arranged regularly within a 400 m × 400 m monitoring plot. Additionally, we estimated the physical and chemical parameters of the same sampling locations. The distribution patterns of the soil mite community and the edaphic parameters were analyzed using a range of geostatistical tools. Moran’s *I* coefficient showed that, during each sampling period, the total abundance of the soil mite communities and the abundance of the dominant mite populations were spatially autocorrelated. The soil mite communities demonstrated clear patchy distribution patterns within the study plot. These patterns were sampling period-specific. Cross-semivariograms showed both negative and positive cross-correlations between soil mite communities and environmental factors. Mantel tests showed a significant and positive relationship between soil mite community and soil organic matter and soil pH only in August. This study demonstrated that in the cornfield, the soil mite distribution exhibited strong or moderate spatial dependence, and the mites formed patches with sizes less than one hundred meters. In addition, in this long-term tillage agroecosystem, soil factors had less influence on the observed pattern of soil mite communities. Further experiments that take into account human activity and spatial factors should be performed to study the factors that drive the spatial distribution of soil microarthropods.

## Introduction

Understanding the spatial distribution of soil fauna is a key topic in community ecology [1]. Soil fauna are important decomposers in agro-ecosystems. These organisms participate in soil biogeochemical cycles and affect the soil’s biological fertility by decomposing organic materials, recycling nutrients and stimulating fungal and bacterial metabolism [2, 3]. Investigating the spatial patterns and decisive factors related to these organisms may provide fundamental data about soil fauna community ecology and establish an important foundation for maintaining biodiversity [4]. The spatial distribution of soil fauna is scale—dependent, and in different ecosystems the organism groups may show different spatial patterns [5]. Some studies have explained the spatial patterns of soil fauna at the regional or local scale (>10^3^m) in forest ecosystem[6, 7]; however, the available information concerning the spatial patterns of soil fauna communities at a small scale (10^1^–10^3^ m) in long-term tillage agro-ecosystems are still insufficient.

On various spatial scales, soil fauna exhibit complicated and structured spatial distribution patterns [8]. On a given scale, the soil fauna distribution is typically not stochastic and may show aggregated or segregated patterns that can be estimated [9]. This behavior may be attributable to different regulatory mechanisms that are active at different spatial scales [10]. Soil organisms are rarely spatially independent at a field scale of <100 m [6]. Some reports, which were based on multi-scale analysis, have shown that soil fauna may exhibit significant spatial correlation, and soil fauna have also been shown to aggregate in patches of 20 cm to dozens of meters in size [6, 11]. A study that investigated soil nematodes within an agro-ecosystem showed a strong aggregated distribution pattern at the field scale [12]. In farmland planted with soybean, the soil mite communities showed clumped distributions at a scale of 5–40 m, with spatial structures. Spatial heterogeneity was a common characteristic of these communities [11]. Thus, multi-scale research that involves spatial analysis is an important method for elucidating the structural characteristics of these communities [9, 13, 14].

In heterogeneous landscapes, environmental factors at the patch and plot scales are generally significant and explain a significant portion of community changes [10, 15]. Different species may have different responses to heterogeneous environments and may thus show dissimilar spatial distributions [16]. Research on the spatial correlation between soil fauna and environmental factors contributes to determining the regulatory mechanisms that control the spatial patterns of soil fauna [17, 18]. For example, soil moisture was demonstrated to be an important factor that affects the spatial patterns of soil mite communities in wetland habitats [19]. In a grassland system, the large-scale spatial distribution pattern exhibited by termites in a 330 m patch was associated with the terrain and vegetation [20]. The spatial distribution pattern of an arthropod species in arable land was shown to depend on the spatial heterogeneity of food resources. Food resources can regulate arthropod species population density [21]. In addition, over time, the spatial distribution of soil fauna is dynamic and more volatile than the spatial distribution patterns of non-biological resources [22]. In an agroecosystem, due to long-term tillage and the planting of a single crop, the ecosystem is homogeneous. Thus, determining whether environmental factors will affect the soil fauna community spatial pattern is important to maintaining diversity of the community.

Autocorrelation is a potential problem in many field sampling studies, as the samples are not independent because samples collected closer to each other are often more similar than samples collected farther from each other. In our study, as soil mites are ideal agents, we chose the soil mite community, which constitutes one of the most important types of organisms found in soil systems. Soil mites typically have low dispersal abilities and affect soil biological fertility [23]. Some studies have revealed that soil mites have spatial structures on the order of dozens of meters and develop an aggregated distribution within an experimental area [24]. When autocorrelation is present in a dataset, specific statistical analyses are required to distinguish between true and false relationships. We chose the geostatistics method to determine the spatial structure of the soil mite community and the degree of autocorrelation [25]. The geostatistical method allows analyses of the complex relationships between soil mite communities and environmental factors. In addition, this type of analysis can also calculate the degree and range of soil mite community spatial dependence and describe the stochastic and structural characteristics of the community’s spatial distributions [26].

Northeast China is an important agriculture region, primarily due to the production of soybean and maize. Black soil (Typic Hapludoll, U.S. Soil Taxonomy) dominates this region [27]. Large-scale cultivation and improper management have resulted in a significant decline of soil biodiversity in black soil, and soil fauna have declined by 40% to 70% in many areas. Protecting soil fauna diversity is challenging [28]. Studying the spatial patterns and maintenance mechanisms of soil fauna in farmland is an effective way to protect soil biodiversity. Biodiversity monitoring plots have provided a useful platform for understanding biological characteristics, spatial distribution patterns and the mechanism of community diversity formation [29]. This method is usually applied in forest ecosystems (http://www.ctfs.si.edu) and has rarely been used in agro-ecosystems. To better investigate cropland biodiversity conservation mechanisms, in this study, a 16 hm^2^ permanent plot was built in black soil cropland to study the community spatial distribution of soil biodiversity.

The aim of the present work is to answer the following questions about a long-term tillage cropland ecosystem. (1) Does the soil mite distribution show spatial dependence, and at what scale does the distribution form a patch pattern? (2) Does this spatial pattern exhibit temporal dynamics or spatial structural stability? (3) What is the relationship between soil mites and edaphic factors, and do edaphic factors affect the spatial patterns of soil mite communities?

## Materials and methods

### Study sites

**Fig 1:**
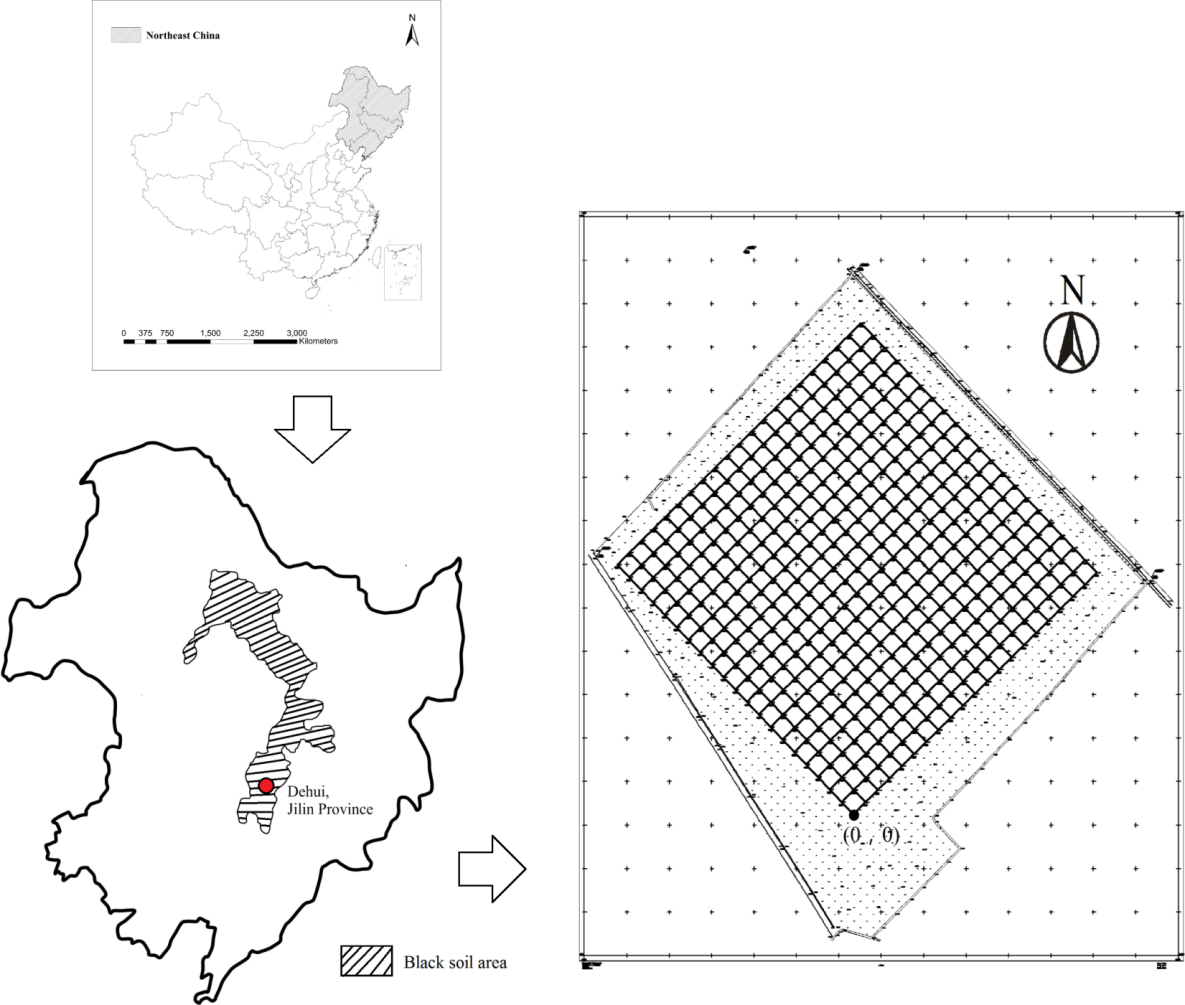
Dimensions of the 16 hm^2^ biodiversity monitoring plot in a black soil cropland. The map on the left shows the administrative divisions of China, and the shaded part of the picture is northeast China. The shaded area in the upper right map is the black soil distribution area. The grid area in the lower right map shows monitoring plots with 20 m intervals, and the point (0, 0) is the coordinate origin of the monitoring plots.

The study was conducted in a 16 hm^2^ permanent cropland plot at the Dehui Agro-ecological Experimental Station of Black Soil (46°36´N, 125°30´E) in the central part of the Song Liao Plain in Jilin Province, China. The region lies within a monsoon climate of medium latitudes and has a warm, humid summer and a cold, dry winter. The climate is characterized by an average annual temperature of 4.4°C and an average annual precipitation of 520 mm. The soil was classified as black soil following the Chinese Soil Classification System, which is equivalent to a Typic Hapludoll in the U.S. Soil Taxonomy [30]. This soil was clay loam-textured, with an average of 36% clay, 24% silt, and 40% sand [27]. In the 0–20 cm soil layer, the pH value is generally 6.5. The site is situated in an area that has been used for corn cultivation for more than 50 years. To prevent the interference of shelter forests and the field road, we established the sampling field in the center of an arable field. We adopted the construction specifications of the Panama Barro Colorado Island (CBI) 50 hm^2^ forest biodiversity monitoring plot [31]. Using a GPS (Global Positioning System) receiver and RTK (Real-Time Kinematic) measurement, the 16 hm^2^ experimental plot was divided into 20 m × 20 m squares with 441 intersection points (Fig 1). Our survey was based on these 40 m × 40 m quadrats with 121 intersection points. The experimental plots have a flat topography; the highest elevation is 174.89 m, and the lowest elevation is 170.0 m. In 2015, the average soil pH value was 7.64, the total percentage of soil organic matter (SOM, %) was 3.02% and the total percentage of soil nitrogen (N, %) was 0.13%.

### Sampling design and laboratory procedures

In the study site, we used each square intersection point as a sampling center. Using an auger, we collected soil mite samples (7 cm in diameter and 10 cm in depth) from the bottom left-hand area of each square. At each sampling point, which was surrounded by an area with a width of 50 cm, we randomly obtained three soil mite samples as a data point. Using the auger and the same sampling method, soil samples (7 cm in diameter and 10 cm in depth) were extracted from an area to the right of the location where the soil mite communities were collected. The field study was conducted on August 1, September 2 and October 4, 2015. We chose these months to sample soil animals in order to ensure that the human disturbance in the farmland was small and that the diversity of soil animals was relative high.

We extracted soil mite communities from the soil cores using the Berlese-Tullgren method [32] and preserved the communities in a 95% alcohol solution. All kind of soil mites were numbered and identified to the species level [33, 34]. We selected the dominant soil mite populations (S1 Table) as the subject of investigation because populations with low abundance do not exhibit significant spatial patterns in our research [35]. Thus, the concept of the total abundance of soil mite communities includes dominant, common and rare populations. The dominant mite species identified were Oribatida, *Incabates major* Aoki, 1970; Oribatida, *Epilohmannia ovata* Aoki, 1961; Oribatida, *Cryptoppia brevisetiger* Wen, Aoki & Wang, 1984; Prostigmata, *Allopygmephorus chinensis* Mahunka, 1975; Mesostigmata, *Gamasellus changbaiensis* Bei & Yin, 1995; Mesostigmata, *Pachylaelaps neoxenillitus* Ma, 1997 (hereafter as M1-M6). The soil samples collected in September were air-dried and sieved to 1 mm (S2 Table). The total percentage of organic carbon (C, %) was determined using Anne’s method, and the percentage of soil organic matter (SOM, %) was then calculated. We used the Kjeldahl method to determine the percentage of total nitrogen (N, %) [36]. The soil pH was measured in deionized water with a soil/solution ratio of 1:5. The soil water content (SWC, %) was determined by weighing the soil before and after drying at 105°C to constant mass [37].

### Statistical analysis

#### Moran’s *I* coefficient

Spatial autocorrelation is an important form of spatial dependence and represents the correlation of the same variables at different spatial positions. The spatial autocorrelation statistics include the spatial positions of the variables and their attributes. We used Moran’s *I* coefficient to measure the autocorrelation of the abundance of soil mite communities [38]. When calculating Moran’s *I* coefficient, we defined 200 m as the active lag distance (half of the length of the study site), the first lag distance as 40 m (as the sampling scale) and the lag interval as 20 m. The value of Moran’s *I* ranges from -1 to 1. When the value of *I* is 0, it indicates that there is no spatial autocorrelation of the soil mites. When the value of *I* is less than 0, it indicates that there is a negative spatial autocorrelation of the soil mites, and when the value of *I* is more than 0, it indicates that there is a positive spatial autocorrelation of the soil mites.

#### Semivariogram analyses

The spatial distribution of the soil organisms was analyzed using geostatistical tools. A semivariogram is a basic statistical tool that is used to express the relationship between the variable and the sample point separation [25, 39]. A semivariogram consists of a plot of half of the average of the squared differences between all pairs of points separated by a specific lag distance [40]. This tool describes the degree of spatial dependence of the variable using a variety of theoretical models.

A standard semivariogram equation is:

$$\gamma \left(h\right)=\frac{1}{2N\left(h\right)}{\sum_{i=1}^{N\left(h\right)}\left[\left(Z\left(\chi_{i}\right)‑Z\left(\chi_{i}+h\right)\right)\right]}^{2}$$ (1)

where *γ*(*h*) is the semivariance function value of *h*; *h* is the separation vector; *N*(*h*) is the number of pairs of data points separated by distance *h*; *x*_*i*_ and (*x*_*i*_ *+ h*) are the location of the point being studied at point *i* and the distance of h from *i*, respectively; *Z* indicates that the semivariogram is computed for variable *Z*.

We chose the best semivariogram model based on the largest R^2^ and the smallest residual sum of squares (RSS) values [41]. In the resulting semivariograms, the nugget (C_0_) implies an unexplained variation that is attributable to either spatial variation at a scale smaller than sampled or measurement errors. When the nugget variance is considered, two sources of variation are involved: human factors (measurement error) and variations within the lag distance used. The range (R) shows the variable size of the soil mites’ distribution and their spatial scale autocorrelation [26]. The ratio of the nugget variance and the structural variance sill (C_0_/(C_0_+C) %) indicates the spatial dependence of the variable [42]. When the proportion is less than 25%, the variable exhibits strong spatial dependence, and the spatial differentiation is determined primarily by structured factors. When the proportion is between 25% and 75%, the variable’s spatial differentiation may be determined by structured or random factors and shows a moderate spatial dependence. Finally, when the proportion exceeds 75%, the variable shows low spatial dependence, and the spatial differentiation is completely attributable to stochastic factors [43]. A nugget effect occurs when the proportion is equal to 1. Thus, no spatial structure exists, and the variable is randomly distributed. The nugget effect corresponds to local variations that may occur at distances shorter than the sampling distance used.

#### Ordinary kriging map

Based on the results from spatial correlation analysis and the best semivariability model, we selected an ordinary kriging map to interpolate the data in the study area. Ordinary kriging is a geographic statistical method that was proposed by Matheron [44]. Ordinary kriging is based on regionalized variable theory and can interpolate values for non-sampled points. This approach provides an optimal interpolation estimate for a given coordinate location. In this study, we simulated the spatial distribution of soil mite communities on a monthly basis to determine the community dynamics in the fields.

$$Z\left(\chi_{0}\right)=\sum_{i=1}^{n}\lambda Z\left(\chi_{i}\right)$$ (2)

Where *Z*(*x*_*0*_) represents the interpolating value for non-sampled points; *Z*(*x*_*i*_) is the interpolating value for points already sampled.

#### Cross-semivariogram analyses

We examined possible spatial correlations between the abundance of soil mite communities and environmental factors via cross-semivariogram analysis [4, 25].

$$\gamma_{AB}\left(h\right)=\frac{1}{2N\left(h\right)}\sum_{i=1}^{N\left(h\right)}\left[Z_{A}\left(\chi_{i}\right)-Z_{A}\left(\chi_{i}+h\right)\right]\left[Z_{B}\left(\chi_{i}\right)-Z_{B}\left(\chi_{i}+h\right)\right]$$ (3)

where *γ*_*AB*_(*h*) is the cross-semivariogram function value of *h* between variables *A* and *B*; *N*(*h*) is the number of pairs of data points separated by distance *h*; and *Z*_*A*_ and *Z*_*B*_ indicate that the attribute values for variables A and B are the same.

Finally, simple Mantel tests [45] were used to assess the links between the spatial patterns of soil mite communities and the spatial patterns of soil parameters during the three-month study period. These tests were performed to determine whether the correlations observed on the cross-semivariograms were true or spurious.

The Moran’s *I* coefficient, semivariogram, and cross-semivariogram analyses were performed using the GS+v.9 (Geostatistics for the Environmental Science) software. The ordinary kriging map was created using the ArcGIS software. The simple mantel test was implemented using ‘mantel’ function in the ‘vegan’ package [46] in R (S1 File), version 3.3.1 (http://www.r-project.org) [47].

## Results

### Spatial autocorrelation of the total soil mite communities

The existence of spatial autocorrelation is the premise of spatial dependence analysis. In total 121 samples from the study site were analyzed. Moran’s *I* coefficient (Table 1) showed that in August, the total abundance of the soil mite communities had a positive spatial autocorrelation within half of the total sample distance, and was significantly correlated at 40–80 meters. In September, the soil mite communities showed positive spatial autocorrelation in the range of 40–60 meters, and were shown significant positive spatial correlation within the half of sample range in October.

**Table 1:** The Moran’s *I* coefficient for the total abundance of the soil mite community in August, September and October.

Month	Separation distance								
	40 m	60 m	80 m	100 m	120 m	140 m	160 m	180 m	200 m
**Aug.**	**0.38** ^**a**^	**0.31**	**0.31**	0.29	0.28	0.29	0.25	0.22	**0.30**
**Sep.**	0.09	0.10	-0.04	-0.08	0.04	-0.02	-0.01	-0.12	0.02
**Oct.**	**0.79**	**0.85**	**0.83**	**0.72**	**0.76**	**0.80**	**0.72**	**0.66**	**0.80**

^a^ When the absolute value of Moran's *I* ranges from 0.3 to 1, it indicates a significant positive spatial autocorrelation of the soil mites (in bold).

### Semivariogram and ordinary kriging maps of total soil mite communities

**Fig 2:**
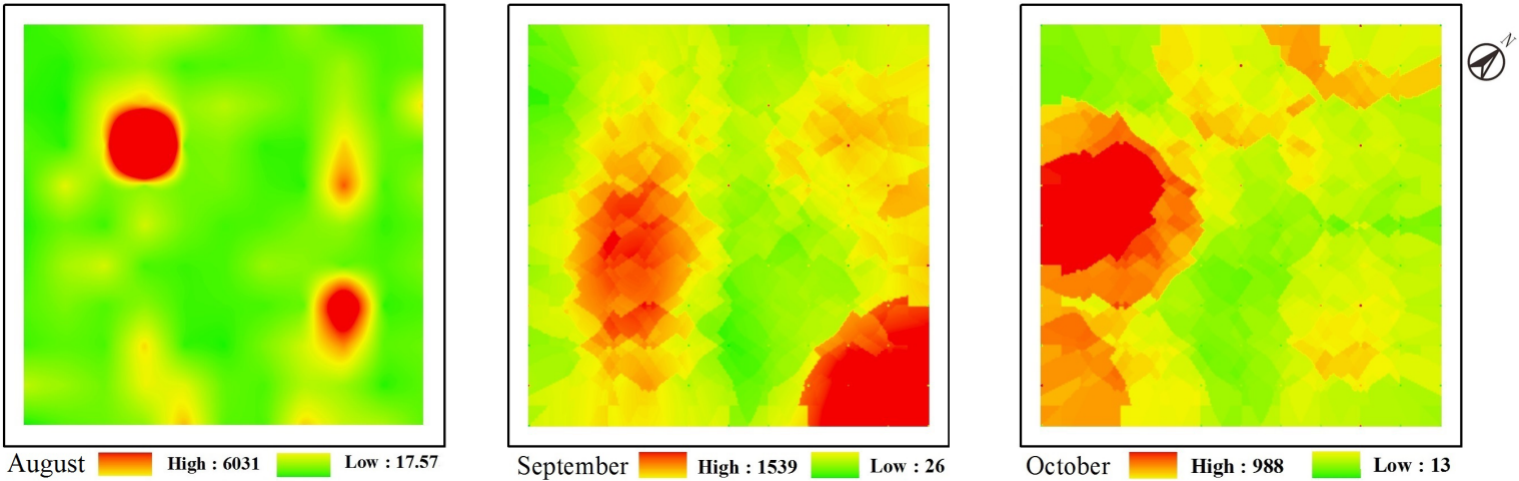
Spatial distribution patterns of the total abundance of soil mite communities. These maps for August, September and October were based on Spherical, Gaussian and Spherical models, respectively.

According to the semivariogram analyses, during each study month, spatial dependence was observed for the total abundance of soil mite communities, revealing a spherical or Gaussian model. The (C_0_/(C_0_+C) %) values of the soil mite communities were 9.18%, 11.53% and 0.55% in August, September, and October, respectively, with all values below 25%. Soil mites were found to form patches with sizes from 47.8 m to 76.9 m. Within the study plot, the ordinary kriging map showed clear patchiness and distribution gradients of the soil mite communities, which generated based on measured data and fitted semivariogram. Moreover, the distribution gradient varied during the months (Fig 2).

## Distribution of dominant soil mite populations

The abundance of the dominant soil mite populations (M1 to M6) showed spatial dependence consistent with an exponential or spherical model (Table 2). In August, the proportion (C_0_/ (C_0_+C) %) of the soil mites were all less than 25% (except for M2), with the formation of patches ranging in size from 68.4 m to 88.2 m. In September, the abundance of M1 showed a nugget effect consistent with a linear model (Table 2). The abundance values of M2 to M6 all showed spatial dependence consistent with exponential, spherical or Gaussian models, and, in September, the communities formed patches from 56.8 m to 84.6 m in size. The proportion of the soil mites (except for M1) were all less than 25%. In October, the dominant mite populations M2 and M4 showed nugget effects consistent with a linear model (Table 2). The proportion of the soil mites were all less than 25%, and the formation of patches that were 56.6 m and 89.7 m in size.

**Table 2:** Theoretical models and corresponding parameters for the semivariograms of dominant soil mite populations in August, September and October.

**Month**	Species ^a^	Variogram model type	Nugget variance (C_0_)	Structural variance sill (C_0_+C)	Proportion [C_0_/(C_0_+C)%]	Range (R)	R^2^	Residual sum of squares (RSS)
**Aug.**	M1	Exponential	0.06	0.72	8.30	73.20	0.74	0.0018
	M2	Spherical	1.103	2.61	42.34	68.40	0.88	0.0098
	M3	Spherical	0.05	1.07	4.38	86.20	0.88	0.0071
	M4	Spherical	0.20	3.32	6.14	77.30	0.84	0.0484
	M5	Exponential	0.09	0.68	13.55	75.30	0.89	0.0006
	M6	Spherical	0.10	1.00	10.28	88.20	0.81	0.0099
**Sep.**	M1	Linear	1.65	1.65	100.0	169.07	0.21	0.0424
	M2	Spherical	0.00	1.68	0.06	56.80	0.67	0.0013
	M3	Exponential	0.15	1.27	11.84	84.60	0.79	0.0065
	M4	Spherical	0.06	2.41	2.28	72.00	0.90	0.0103
	M5	Gaussian	0.03	0.74	4.19	57.33	0.57	0.0044
	M6	Exponential	0.08	1.50	5.02	75.30	0.97	0.0008
**Oct.**	M1	Spherical	0.00	1.14	0.09	56.60	0.05	0.0226
	M2	Linear	1.87	1.87	100.00	169.06	0.42	0.0440
	M3	Exponential	0.04	1.11	3.98	71.70	0.71	0.0053
	M4	Linear	2.17	2.17	100.00	169.06	0.33	0.0644
	M5	Exponential	0.00	1.21	0.17	89.70	0.76	0.0103
	M6	Exponential	0.01	1.20	1.09	87.90	0.78	0.0083

^a^ M1, Oribatida, *Incabates major* Aoki, 1970; M2, Oribatida, *Epilohmannia ovata* Aoki, 1961; M3, Oribatida, *Cryptoppia brevisetiger* Wen, Aoki & Wang, 1984; M4, Prostigmata, *Allopygmephorus chinensis* Mahunka, 1975; M5, Mesostigmata, *Gamasellus changbaiensis* Bei & Yin, 1995; M6, Mesostigmata, *Pachylaelaps neoxenillitus* Ma, 1997.

### Relationships between total soil mite communities and soil parameters

**Fig 3:**
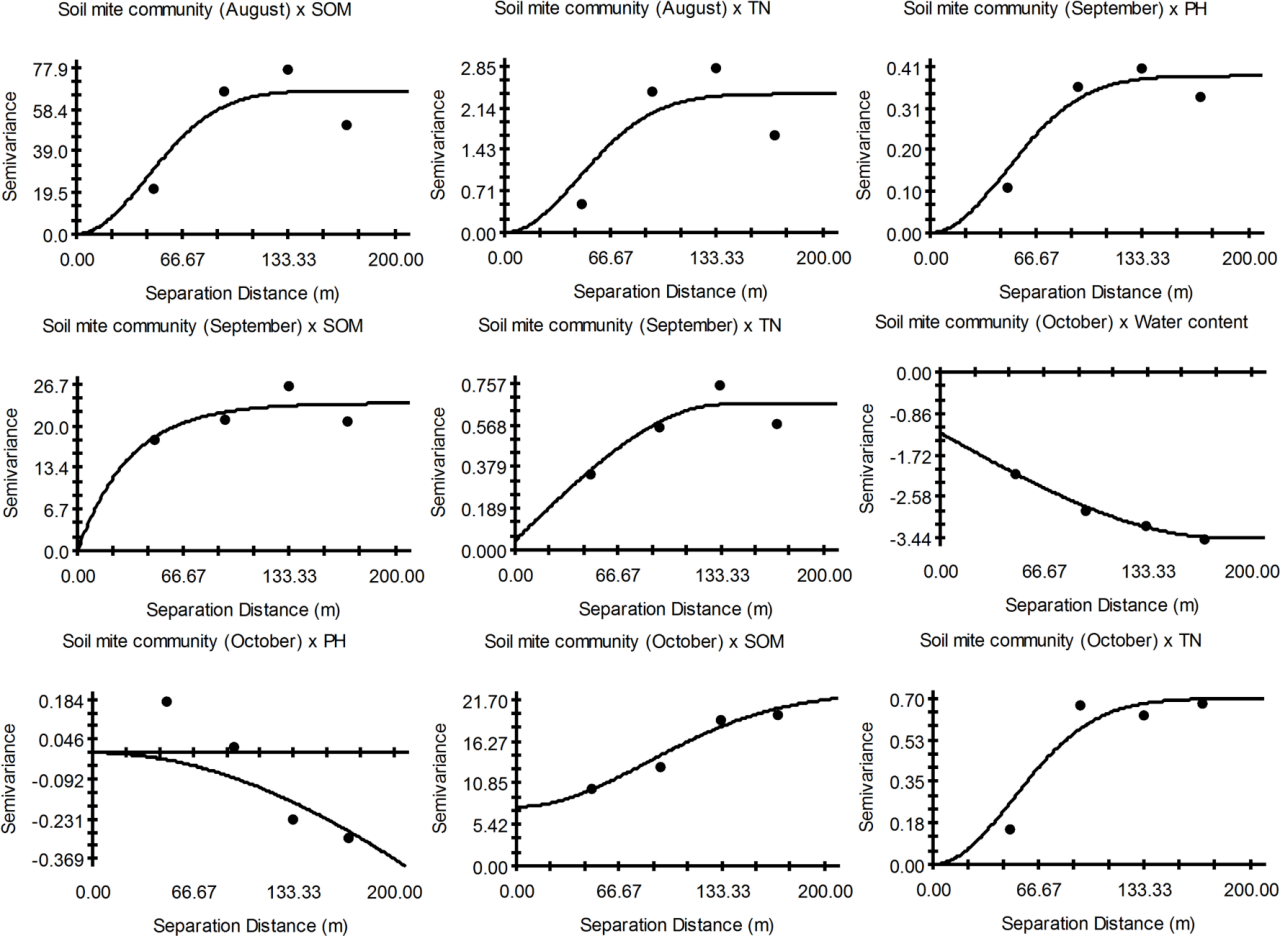
Cross-semivariograms showing relationship between the total abundance of soil mite community and soil parameters in August, September and October. SWC, soil water content (%); SOM, the percentage of soil organic matter (%); and TN, the percentage of total nitrogen (%).

The cross-semivariograms revealed that the total abundance of soil mites and soil parameters exhibited clear positive or negative relationships (Fig 3). In August, the soil mite community showed a multi-scale positive relationship with SOM (%) and TN (%), whereas in September, the soil mite community exhibited a continuous positive correlation with soil pH, SOM (%) and TN (%). In October, the abundance of the soil mite community correlated negatively with SWC (%) and pH, and had positive correlated with SOM (%) and TN (%).

The Mantel test showed that in August there was a significant positive relationship between SOM (%) and soil mite communities, as well as the relationship between pH and soil mite communities (S3 Table).

### Relationships between dominant populations of soil mites and soil parameters

The soil water content in August showed a continuous negative relationship with the dominant soil mite species M1, M2 and M5 but positive relationships with M3 and M6, as their patch sizes varied from 48 m to 169 m (Table 3). Except for when the patch size was 92 m– 132 m, M4 was positively related to the soil water content. When the patch size was 48 m– 169 m, the soil pH, SOM and TN exhibited negative relationships with M1 and continuous positive relationships with M2, M3, M4, M5, and M6. In September and October, the abundance of soil mites showed complex positive or negative spatial correlations with edaphic factors on multiple scales. The results of the simple Mantel test showed that the spatial relationships between the dominant soil mite populations and environmental factors were not significant during the three study months.

**Table 3:** The spatial relationships between the dominant soil mite populations and soil parameters in August, September and October.

month	species^a^	SWC(%) ^b^				pH ^b^				SOM(%) ^b^				TN(%) ^b^			
		48m	92m	132m	169m	48m	92m	132m	169m	48m	92m	132m	169m	48m	92m	132m	169m
**Aug.**	M1	-^c^	-	-	-	-	-	-	-	-	-	-	-	-	-	-	-
	M2	-	-	-	-	+^c^	+	+	+	+	+	+	+	+	+	+	+
	M3	+	+	+	+	+	+	+	+	+	+	+	+	+	+	+	+
	M4	-	+	+	-	+	+	+	+	+	+	+	+	+	+	+	+
	M5	-	-	-	-	+	+	+	+	+	+	+	+	+	+	+	+
	M6	+	+	+	+	+	+	+	+	+	+	+	+	+	+	+	+
**Sep.**	M1	+	+	+	+	+	+	+	+	-	+	+	+	+	+	+	+
	M2	+	+	+	+	+	+	+	+	+	+	+	+	-	-	+	-
	M3	-	+	+	+	-	+	+	+	+	+	+	+	+	+	+	+
	M4	+	+	+	+	+	+	+	+	+	+	+	+	-	+	+	+
	M5	-	+	-	+	+	+	+	+	+	+	+	+	+	+	+	+
	M6	+	+	+	+	+	+	+	+	+	+	+	+	-	+	+	+
**Oct.**	M1	+	+	+	+	+	+	-	-	-	-	-	-	-	-	-	-
	M2	-	-	-	-	-	-	-	-	+	+	+	+	+	+	+	+
	M3	-	-	-	-	+	+	+	+	+	+	+	+	+	+	+	+
	M4	-	-	-	-	+	+	-	-	+	+	+	+	+	+	+	+
	M5	-	-	-	-	+	-	+	+	+	+	+	+	+	+	+	+
	M6	-	-	-	-	-	-	-	-	-	+	+	+	-	+	+	+

^a^ M1, Oribatida, *Incabates major* Aoki, 1970; M2, Oribatida, *Epilohmannia ovata* Aoki, 1961; M3, Oribatida, *Cryptoppia brevisetiger* Wen, Aoki & Wang, 1984; M4, Prostigmata, *Allopygmephorus chinensis* Mahunka, 1975; M5, Mesostigmata, *Gamasellus changbaiensis* Bei & Yin, 1995; M6, Mesostigmata, *Pachylaelaps neoxenillitus* Ma, 1997.

^b^ SWC, soil water content (%); pH, soil pH; SOM, the percentage of soil organic matter (%); and TN, the percentage of total nitrogen (%).

^c^+, The abundance of soil mite populations and environmental factors are positive correlation in this spatial scales; -, The abundance of soil mite populations and environmental factors are negative correlation in this spatial scales.

## Discussion

The horizontal distribution of soil fauna is complex and structured at different spatial scales [14]. Exploring how community distribution patterns change across spatial and temporal scales is very important in community ecology [48]. We used geostatistical methods to describe the spatial distribution patterns of soil mites and to elucidate the relationships between soil mites and environmental factors. The Moran’s *I* coefficient demonstrated that the total soil mite communities and the dominant soil mite populations are spatially autocorrelated and reflect a clear spatial structure. Soil mites are small and wingless animals that live in soil ecosystems [49]. Relevant studies have shown that dispersal limitation should be an important driver for soil mites’spatial distribution [50, 51]. Soil mite species were found to be severely dispersal limited even at an isolated distance as short as a few centimeters, which facilitates an aggregated spatial distribution and significant spatial autocorrelation [52]. Moreover, soil mites have considerable passive dispersal abilities. Diffusion barriers, such as lack of continuous inter-connectance among soil pores might affect the active movements of some soil mite species [53]. Species richness was also an important factor that affected the spatial autocorrelation. In addition, the spatial autocorrelation of the species may be affected by climate conditions, soil types, interspecific competition, home-range size and other factors [54]. In our study, the spatial autocorrelation analysis of distance constraints suggested that the spatial autocorrelation of soil mites may be subject to small-scale interference and may play an important role in spatial autocorrelation localization. This change reflects the complexity of the soil mite spatial structure and the diversity of the influencing factors.

Semivariogram analysis revealed that the soil mite populations were aggregated in patches across a few dozen meters and followed spherical or exponential models. These findings are consistent with the results of previous studies, in which the soil fauna were usually observed to be highly aggregated in hot spots and to form spatial structures at various spatial scales [24]. The proportion demonstrated that, in each month, the spatial distributions of the soil mite communities were determined primarily by structured factors only or by both structured and stochastic factors. Meanwhile, nugget effects were detected for the dominant soil mite populations in September and October, which showed weakly spatial dependent. This might be a result of sampling errors or spatial variability within the minimum distance interval. In addition, the soil parameters strongly or moderately depends on spatial structure, with patches that ranged in size from 64 m to 119 m (S4 Table). A comparison of soil mite communities and soil parameters revealed that their spatial distribution patterns changed across temporal scales.

The ordinary kriging map indicated that the soil mite communities presented clear patchy distribution patterns, and the direction along the ridge (northwest-southeast) showed a regular distribution trend. Over time, the spatial distribution range of the soil mite community gradually expanded, and the shape and size of the plaques changed. The formation of these plaques may be related to the micro-terrain, food resource gradients [2] or biological competition [55]. Soil moisture content, surface temperature and other non-deterministic seasonal changes that affect soil mites, will also impact the spatial distribution of the communities. In agroecosystems, agricultural management activities, such as fertilization, irrigation, weeding and pest control, are also important factors that affect the spatial distribution of soil mites [49].

According to the cross-semivariograms analysis, during each study month, the soil mite communities and most of the soil mite populations showed complex positive or negative relationships with soil factors. In addition, the simple Mantel test was only significant and positive for the relationship between SOM (%) and the soil mite community in August. This finding indicates the existence of a spurious spatial correlation between the soil mite community and environmental factors. This association may be affected by the spatial patterns of species and may not indicate a true interrelation. Soil organisms often show aggregated patterns of abundance at the mesoscale (a few meters up to 100 m). At these spatial scales, local community distribution is often due to abiotic factors [56]. The niche differences between species in, e.g., the tolerance to abiotic conditions such as soil pH and soil moisture will determine the community composition [4]. However, in our study, there was no significant spatial correlation between soil organic matter, soil water content, soil pH, total nitrogen and soil mite communities, except August. Some study suggested biotic factors, e.g., species interactions and vegetation variations, may also affect local community distribution [5, 10]. For instance, the study by Bonari et al. [51] showed that in a mediterranean stone pine forest the soil features were not of primary importance for soil mite community assemblages. The vegetation cover can be considered a driver of mite communities. Gao et al. [11] also studied the spatial relationships between soil mites and environmental factors in different months and found that soil mite communities and environmental factors exhibited positive or negative spatial relationships, but those correlations did not reach significance. Our findings cannot deny the existence of a spatial correlation between environmental factors and soil mites because the ecological processes that occur at different spatial scales could differ. This possibility makes a multi-scale analysis of spatial relationships very important [57]. Gutiérrez-López et al. [26] investigated various spatial correlations between soil species and environmental factors on different spatial scales and confirmed this idea. Kaenko [58] revealed that the soil animal community and the soil water content were positively related; however, under multi-scale conditions, this relationship was uncertain. Thus, greater complexity and uncertainty of the factors can affect the results. In addition, the environmental factor-related data (soil moisture content, pH, organic matter and total nitrogen) measured in this study were obtained in a single year. However, the results of a long-term sequence may better confirm the relationship between environmental factors and soil mite communities.

Many studies have questioned the manner in which factors influence the distribution of soil organisms. According to niche and neutral theories in community ecology, the composition of species assemblages can be explained by three processes: environmental filtering (i.e., physical or chemical, climatological and geomorphological factors), biotic interactions (i.e., competition) and dispersal limitation [59, 60]. Related research has addressed niche theory and neutral theory as important mechanisms that affect the regulation of soil fauna spatial patterns. However, the regulatory mechanisms that act at different spatial scales may differ [9]. At the scale used for our study, we found that the distributions of soil mite communities in each month were determined by spatial or stochastic factors, which showed strongly or moderately spatial depends. And the environmental variables have shown little variation and have low influence on soil mite community. This finding suggests that other factors, e.g., species interactions, differences in home range and dispersal limitation may also affect local community distribution. A related study [11] suggested that during the fine-scale (5 m) structuring of a soil mite community in farmland, both dispersal limitation and environmental filtering were important drivers, whereas biotic interactions had less influence on the observed pattern. Ingimarsdo´ttir et al [61] also found on the nunataks within the glacier, local environmental factors were important in structuring the soil collembolans and oribatids community assembly, but the effects of dispersal cannot ignored. Meanwhile, nugget effects were detected for the main soil mite species in our study, the results show that the spatial processes that occur at a scale of less than 40 m cannot be neglected. In our study, we found that edaphic factors had less influence on the observed patterns of soil mite communities in a long-term tillage cornfield. Thus, further experiments should be performed to study the factors that drive the spatial distribution of soil microarthropods while taking into account human activity and spatial factors.

## Conclusions and future prospects

This study demonstrated that soil mite communities exhibit spatial variability that changes over time and that both structured and stochastic factors exert regulatory effects. A confluence of multiple interacting processes probably affects the distributions of different soil groups and their relationships. Within the study area, the soil mite community and environmental factors exhibited both positive and negative correlations, and environmental factors had less influence on the observed patterns of soil mite communities. This study only showed the spatial distribution patterns of soil mites and the spatial correlations of these patterns with environmental factors at the 400 m × 400 m scale. Thus, further multi-scale experiments are necessary in our monitoring plot. Therefore, in future studies, multi-scale, long-term and multi-variable experiments (i.e., human disturbance and the impact of agricultural management activities) must be performed to fully understand the processes that affect the specific patterns observed in the present study.

## Supporting information

S1 Table Species and individuals of dominant soil mite populations in August, September and October (n = 121 samples). (PDF)

S2 Table Characteristics of soil parameters (n = 121 samples). (PDF)

S3 Table Simple Mantel test of total soil mite community dissimilarity against soil parameters in August, September and October (999 permutations). (PDF)

S4 Table Theoretical models and corresponding parameters for the semivariograms of soil parameters. (PDF)

S1 File R code for the simple mantel test. (PDF)
