# Supplementary material for: Spatial distribution patterns of soil mite communities and their relationships with edaphic factors in a 30-year tillage cornfield in northeast China
Source: PLoS One. 2018 Jun 28;13(6):e0199093. doi: 10.1371/journal.pone.0199093 (PMC6023156; doi:10.1371/journal.pone.0199093)
Supplement: S1 Table — (PDF) [file pone.0199093.s001.pdf]

S1 Table. Species and individuals of dominant soil mite populations in August, September and October (n=121 samples).

Species [a]	August			September			October		
	Individuals	Percentage of total mite (%)	CV (%) [b]	Individuals	Percentage of total mite (%)	CV (%)	Individuals	Percentage of total mite (%)	CV (%)
**M1**	512	1.37	1.13	1366	5.23	1.13	1016	4.62	1.22
**M2**	9420	25.21	2.29	5754	22.03	1.06	7192	32.68	1.42
**M3**	6779	18.14	1.14	6551	25.08	1.03	4842	22.00	1.14
**M4**	11485	30.74	4.66	5780	22.13	2.29	5435	24.70	2.31
**M5**	495	1.32	1.02	1832	7.01	0.77	899	4.09	2.25
**M6**	1719	4.60	1.28	1577	6.04	1.98	696	3.16	3.48

[a] M1, Oribatida, *Incabates major* Aoki, 1970; M2, Oribatida, *Epilohmannia ovata* Aoki, 1961; M3, Oribatida, *Cryptoppia brevisetiger* Wen, Aoki & Wang, 1984; M4, Prostigmata, *Allopygmephorus chinensis* Mahunka, 1975; M5, Mesostigmata, *Gamasellus changbaiensis* Bei & Yin, 1995; M6, Mesostigmata, *Pachylaelaps neoxenillitus* Ma, 1997.

[b] CV, coefficient of variation.
