# Supplementary material for: Spatial distribution patterns of soil mite communities and their relationships with edaphic factors in a 30-year tillage cornfield in northeast China
Source: PLoS One. 2018 Jun 28;13(6):e0199093. doi: 10.1371/journal.pone.0199093 (PMC6023156; doi:10.1371/journal.pone.0199093)
Supplement: S2 Table — (PDF) [file pone.0199093.s002.pdf]

S2 Table. Characteristics of soil parameters (n=121 samples)

soil parameters [a]	month	Mean	SD [b]	CV(%) [c]
**SWC (%)**	Aug.	21.63	5.77	0.27
	Sep.	19.75	1.93	0.10
	Oct.	24.39	9.15	0.38
**pH**		7.64	0.21	0.03
**SOM (%)**		3.02	0.41	0.14
**TN (%)**		0.13	0.02	0.13

[a] SWC, soil water content (%); pH, soil pH; SOM, the percentage of soil organic matter (%); and TN, the percentage of total nitrogen (%).

[b] SD, standard deviation.

[c] CV, coefficient of variation.
