# Supplementary material for: Spatial distribution patterns of soil mite communities and their relationships with edaphic factors in a 30-year tillage cornfield in northeast China
Source: PLoS One. 2018 Jun 28;13(6):e0199093. doi: 10.1371/journal.pone.0199093 (PMC6023156; doi:10.1371/journal.pone.0199093)
Supplement: S3 Table — (PDF) [file pone.0199093.s003.pdf]

S3 Table. Simple Mantel test of total soil mite community dissimilarity against soil parameters in August, September and October (999 permutations).

month	SWC(%) [a]		pH [a]		SOM(%) [a]		TN(%) [a]	
	R	***P***	***R***	***P***	***R***	***P***	***R***	***P***
**Aug.**	-0.06	0.87	0.09	0.03*	0.11	0.02*	0.06	0.13
**Sep.**	0.07	0.07	0.00	0.46	0.03	0.23	0.00	0.47
**Oct.**	-0.04	0.73	-0.01	0.58	0.01	0.57	0.01	0.42

[a] SWC, soil water content (%); pH, soil pH; SOM, the percentage of soil organic matter (%); and TN, the percentage of total nitrogen (%); *p<0.05.
