# Supplementary material for: Spatial distribution patterns of soil mite communities and their relationships with edaphic factors in a 30-year tillage cornfield in northeast China
Source: PLoS One. 2018 Jun 28;13(6):e0199093. doi: 10.1371/journal.pone.0199093 (PMC6023156; doi:10.1371/journal.pone.0199093)
Supplement: S4 Table — (PDF) [file pone.0199093.s004.pdf]

S4 Table. Theoretical models and corresponding parameters for the semivariograms of soil parameters

soil parameters [a]	month	Variogram model type	Nugget variance (C_0)	Structural variance sill (C_0+C)	Proportion [C_0/(C_0+C) %]	Range (R)	R^2	Residual sum of squares (RSS)
**SWC (%)**	Aug.	Spherical	6.10	39.50	15.44	68.30	0.13	83.30
	Sep.	Spherical	0.03	3.19	1.00	64.00	0.40	0.08
	Oct.	Exponential	33.40	122.10	27.36	119.40	0.80	109.00
**pH**		Exponential	0.003	0.04	6.35	75.90	0.48	<0.0001
**SOM (%)**		Exponential	0.00	0.16	0.06	101.00	0.83	<0.0001
**TN (%)**		Spherical	0.00	0.00	32.00	80.30	0.92	<0.0001

[a] SWC, soil water content (%); pH, soil pH; SOM, the percentage of soil organic matter (%); and TN, the percentage of total nitrogen (%).
