# Supplementary material for: Spatial distribution patterns of soil mite communities and their relationships with edaphic factors in a 30-year tillage cornfield in northeast China
Source: PLoS One. 2018 Jun 28;13(6):e0199093. doi: 10.1371/journal.pone.0199093 (PMC6023156; doi:10.1371/journal.pone.0199093)
Supplement: S1 File — (PDF) [file pone.0199093.s005.pdf]

S1 File. R code for the simple mantel test

```
## Is the abundance of soil mite related to soil parameters?

Library (vegan)
#Reading the August Species Matrix and the Soil Parameters Matrix
spe<-read.csv("201508M.csv",header=TRUE)
env<-read.csv("env.8.csv",header=TRUE)
# Bray-Curtis
veg.dist <- vegdist(spe)
env.dist <- vegdist(scale(env), "euclid")
mantel(veg.dist, env.dist)
mantel(veg.dist, env.dist, method="spear")


#Reading the September Species Matrix and the Soil Parameters Matrix
spe<-read.csv("201509M.csv",header=TRUE)
env<-read.csv("env.9.csv",header=TRUE)
# Bray-Curtis
veg.dist <- vegdist(spe)
env.dist <- vegdist(scale(env), "euclid")
mantel(veg.dist, env.dist)
mantel(veg.dist, env.dist, method="spear")


#Reading the October Species Matrix and the Soil Parameters Matrix
spe<-read.csv("201510M.csv",header=TRUE)
env<-read.csv("env.10.csv",header=TRUE)
# Bray-Curtis
veg.dist <- vegdist(spe)
env.dist <- vegdist(scale(env), "euclid")
mantel(veg.dist, env.dist)
mantel(veg.dist, env.dist, method="spear")
```
